# Gastroesophageal reflux leads to esophageal cancer in a surgical model with mice

**DOI:** 10.1186/1471-230X-9-59

**Published:** 2009-07-23

**Authors:** Jing Hao, Ba Liu, Chung S Yang, Xiaoxin Chen

**Affiliations:** 1 Susan Lehman Cullman Laboratory for Cancer Research, Department of Chemical Biology, Ernest Mario School of Pharmacy, Rutgers, The State University of New Jersey, 164 Frelinghuysen Road, Piscataway, NJ 08854, USA; 2 Cancer Research Program, Julius L. Chambers Biomedical/Biotechnology Research Institute, North Carolina Central University, 700 George Street, Durham, NC 27707, USA

## Abstract

**Background:**

Esophago-gastroduodenal anastomosis with rats mimics the development of human Barrett's esophagus and esophageal adenocarcinoma by introducing mixed reflux of gastric and duodenal contents into the esophagus. However, use of this rat model for mechanistic and chemopreventive studies is limited due to lack of genetically modified rat strains. Therefore, a mouse model of esophageal adenocarcinoma is needed.

**Methods:**

We performed reflux surgery on wild-type, *p53*^*A*135*V *^transgenic, and *INK4a/Arf*^+/- ^mice of A/J strain. Some mice were also treated with omeprazole (1,400 ppm in diet), iron (50 mg/kg/m, *i.p*.), or gastrectomy plus iron. Mouse esophagi were harvested at 20, 40 or 80 weeks after surgery for histopathological analysis.

**Results:**

At week 20, we observed metaplasia in wild-type mice (5%, 1/20) and *p53*^*A*135*V *^mice (5.3%, 1/19). At week 40, metaplasia was found in wild-type mice (16.2%, 6/37), *p53*^*A*135*V *^mice (4.8%, 2/42), and wild-type mice also receiving gastrectomy and iron (6.7%, 1/15). Esophageal squamous cell carcinoma developed in *INK4a/Arf*^+/- ^mice (7.1%, 1/14), and wild-type mice receiving gastrectomy and iron (21.4%, 3/14). Among 13 wild-type mice which were given iron from week 40 to 80, twelve (92.3%) developed squamous cell carcinoma at week 80. None of these mice developed esophageal adenocarcinoma.

**Conclusion:**

Surgically induced gastroesophageal reflux produced esophageal squamous cell carcinoma, but not esophageal adenocarcinoma, in mice. Dominant negative *p53 *mutation, heterozygous loss of *INK4a/Arf*, antacid treatment, iron supplementation, or gastrectomy failed to promote esophageal adenocarcinoma in these mice. Further studies are needed in order to develop a mouse model of esophageal adenocarcinoma.

## Background

Esophageal adenocarcinoma is a rising malignancy in western world in the past 30 years, and now exceeds the incidence of esophageal squamous cell carcinoma [1]. It is generally recognized that gastroesophageal reflux disease (GERD) and Barrett's esophagus are the major risk factors of esophageal adenocarcinoma [2]. Barrett's esophagus is a metaplastic lesion of esophageal squamous epithelium adapted to GERD. The risk of esophageal adenocarcinoma in patients with Barrett's esophagus is around 0.5% per year [3]. Even with extensive treatment, the 5-year survival rate of esophageal adenocarcinoma is still around 20% [4]. Therefore, it is important to better understand the underlying mechanisms of this disease.

Both genetic and environmental factors may contribute to the development of esophageal adenocarcinoma. Along with the progression of metaplasia, dysplasia and adenocarcinoma, *p53 *gene mutation and protein accumulation were frequently detected [5,6]. It has been reported that *p16*^*INK*4*a *^was frequently silenced by promoter hypermethylation in esophageal adenocarcinoma [7,8]. *p14*^*Arf *^was also silenced by hypermethylation in some cases, yet to a less extent compared with *p16*^*INK*4*a *^[7]. Among the environmental factors, long-term antacid therapy might promote esophageal adenocarcinoma by producing hypergastrinemia and promoting bile acid-induced mutagenesis in a neutral pH environment [9,10]. Gastrectomy might contribute to the development of esophageal adenocarcinoma by inducing GERD [11]. Iron supplementation promoted carcinogenesis through oxidative stress as we have shown in the surgical models with rats [12].

Animal models are great tools for research on human diseases. An ideal animal model should recapitulate the disease in humans in etiology, pathogenesis, and molecular features. Currently, the most popular animal models of esophageal adenocarcinoma are surgical models with rats [12-15]. Several strains of rats, e.g., Sprague-Dawley, F344, Wistar, have been used to generate esophageal adenocarcinoma [14,16,17]. Various surgical procedures, such as esophagojejunostomy, esophagoduodenal anastomosis, and esophagogastroduodenal anastomosis, have been reported by us and others [12,14,15]. Both carcinogenesis and chemoprevention studies using rat models lead us to better understanding of esophageal adenocarcinoma and possible preventive strategies [18-23]. However, the use of rat models in mechanistic studies has been limited due to lack of genetically modified rat strains.

Mouse esophagus is very similar to rat esophagus in histology. Several mouse models of esophageal adenocarcinoma have been reported. Duncan *et al*. reported an *E1A/E1B *transgenic mouse model [24]. The advantage of this model was that mice developed adenocarcinoma at the squamocolumnar junction without surgery or carcinogen treatment. However, these mice can not be bred to keep a stable line. Fein *et al*. reported a *p53 *knockout mouse model with gastrectomy and esophagojejunostomy [25]. Out of twelve *p53 *knockout mice, 4 survived after 24 weeks of observation. Two of them had esophageal adenocarcinoma and another one had squamous cell carcinoma. In our study using *p53 *knockout mice, 28 of 32 operated mice died within 20 weeks after surgery and most within 8 weeks, due to spontaneous lymphomas or sarcomas. All of the 4 mice that survived 20 weeks after surgery developed visible tumors of esophageal adenocarcinoma (unpublished data). Therefore, due to the short life span of *p53 *knockout mice, application of this model is very limited. Another research group reported a mouse model of esophageal adenocarcinoma induced with esophagojejunostomy and a carcinogen, N-methyl-N-benzyl nitrosamine. Both wild-type and *p27 *knockout mice of Swiss-Webster strain developed esophageal adenocarcinoma and squamous cell carcinoma, yet at a low frequency. With *p27 *knockout, the incidence of esophageal adenocarcinoma was as low as 23.3% [26,27].

In this study, we aimed to develop a mouse model of esophageal adenocarcinoma with a well-established surgical procedure, which successfully induced esophageal adenocarcinoma in rats [12]. *p53*^*A*135*V *^mice carrying a dominant negative mutation of *p53 *gene, which have a longer life span and compromised *p53 *function [28], were used to enhance carcinogenesis. *INK4a/Arf*^+/- ^mice were also included to test the potential effect of down-regulation of *p16*^*INK*4*a *^and *p14*^*Arf *^genes on carcinogenesis. Besides, the potential roles of gastric acid and iron supplementation in carcinogenesis were examined by combining surgery with acid suppression and/or iron supplementation.

## Methods

### Animal breeding and maintenance

Wild-type A/J mice were obtained from the Jackson Laboratory (Bar Harbor, ME) as breeders. Two genetically modified mouse strains were obtained from Dr. Ming You's group at Washington University School of Medicine: [1] A/J mice carrying three copies of an *Ala-135-Val p53 *mutant transgene [28]; [2] A/J mice with heterozygous knockout of *INK4a/Arf *[29]. PCR genotyping was performed as described elsewhere [29].

We bred 151 male and 62 female wild-type mice, 36 male and 42 female *p53*^*A*135*V *^transgenic mice, and 24 male and 5 female *INK4a/Arf*^+/- ^mice for this study in our animal facility. These mice were housed 10 per cage in plastic cages with hardwood bedding and dust covers, in a HEPA-filtered, environmentally controlled room (24 ± 1°C, 12/12 h light/dark cycle). Animals were given lab chow before surgery. Solid food was withdrawn for one day after surgery. All mice were on AIN93M diet after surgery, except that Group E received AIN93M diet supplemented with 1,400 ppm omeprazole. All the diets were made by Research Diets, Inc. (New Brunswick, NJ) once every month and kept at 4°C until use.

### Surgical procedures

**Figure 1:**
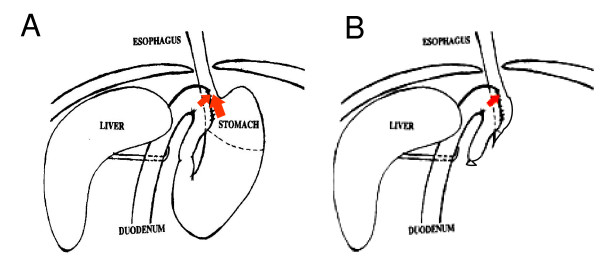
**Surgical produces producing gastroesophageal reflux**. (A) esophagogastroduodenal anastomosis; (B) esophagogastroduodenal anastomosis plus gastrectomy.

Six- to eight-week-old A/J mice were administered anesthetics pre-mixed in normal saline (80 mg/kg ketamine and 12 mg/kg xylazine, *i.p*.). Esophagogastroduodenal anastomosis was performed through an upper midline incision. Two 0.5 cm incisions were made on the esophagus and the duodenum on the anti-mesenteric border, and then were anastomosed together with accurate mucosal to mucosal opposition. Total gastrectomy was performed on some mice following the reflux procedure (Figure 1). These surgical procedures were approved by the Animal Care and Facilities Committee at Rutgers University (protocol no. 94-017).

After surgery, mice were divided into 6 groups: Group B (reflux surgery, 49 male and 52 female wild-type mice); Group C (reflux surgery, 36 male and 42 female *p53*^*A*135*V *^transgenic mice); Group D (reflux surgery, 24 male and 5 female *INK4a/Arf*^+/- ^mice); Group E (reflux surgery plus omeprazole and iron, 36 male wild-type mice); Group F (reflux surgery plus iron, 30 male wild-type mice); and Group G (reflux surgery plus gastrectomy and iron, 26 male wild-type mice). One group of wild-type mice (10 male and 10 female) were used as non-operated control (Group A) (Table 1).

**Table 1:** Development of metaplasia and squamous cell carcinoma in mouse esophagus due to gastroesophageal reflux

			Week 20	Week 40		Week 80
					
Group	Genotype	Treatment	Metaplasia	Metaplasia	Squamous cell carcinoma	Squamous cell carcinoma
A	Wild-type	Non-operated control	0/10	0/10	0/10	-
B	Wild-type	Reflux surgery and 50 mg/kg/m Fe, *i.p*. (Wk 41 to 80)	1/20 (5%)	6/37 (16.2%)	0/37	12/13 (92.3%)
C	*p53*^*A*135*V*^	Reflux surgery	1/19 (5.3%)	2/42 (4.8%)	0/42	-
D	*INK4a/Arf*^+/-^	Reflux surgery	0/10	0/14	1/14 (7.1%)	-
E	Wild-type	Reflux surgery, omeprazole (1,400 ppm) and 50 mg/kg/m Fe, *i.p*. (Wk 21 to 40)	-	0/11	0/11	
F	Wild-type	Reflux surgery and 50 mg/kg/m Fe, *i.p*. (Wk 2 to 40)	-	0/22	0/22	-
G	Wild-type	Reflux surgery, gastrectomy and 50 mg/kg/m Fe, *i.p*. (Wk 2 to 40)	-	1/14 (6.7%)	3/14^a^ (21.4%)	-

^a ^Compared with Group B (P < 0.05)

Iron dextran (50 mg Fe/kg/month; Henry Schein, Melville, NY) was administered to mice of 4 groups through *i.p*. injection. Group F and Group G received iron supplementation starting from 2 weeks after surgery. Group E received iron supplementation from 20 to 40 weeks after surgery and Group B from 40 to 80 weeks after surgery.

All mice were euthanized by CO_2_. Esophagus was removed, opened longitudinally, and fixed in 10% buffered formalin for 24 h, and then transferred to 80% ethanol. The formalin-fixed esophagi were Swiss-rolled, processed and embedded in paraffin. Serial sections (5 μm) were mounted onto glass slides and used for histopathological analysis.

### Histopathological analysis and immunostaining

H&E staining was performed on slides (No. 1 and 30) for diagnosis. Metaplasia was diagnosed when mucin-producing cells were observed in the squamous epithelium of mouse esophagus as confirmed by Alcian blue staining. Squamous dysplasia was diagnosed when squamous epithelial cells lost maturation and orientation with epithelial disorganization. Esophageal squamous cell carcinoma was diagnosed when squamous epithelial cells lost their normal orientation, had a high nucleus-to-cytoplasm ratio, and were heterochromatic, and dysplastic cells broke basement membrane and invaded into lamina propria [30].

Tissue sections of wild-type mice (Group B) and *p53*^*A*135*V *^transgenic mice (Group C) were stained for p53 protein in the esophagus. Paraffin sections were dewaxed in xylene, and rehydrated in a gradient of ethanol to distilled water. After quenching endogenous peroxidase activity with 3% hydrogen peroxide, tissue sections were incubated in normal horse serum to minimize non-specific binding. A polyclonal p53 antibody (Cat# NCL-p53-CM5p, Vision BioSystems Inc., Fremont, CA, 1:500) was applied at 4°C overnight. Tissue sections were then incubated with secondary biotin conjugated antibody at room temperature for 30 minutes. An avidin-biotin peroxidase complex (Vector Laboratories, Burlingame, CA) was then applied, and the staining was visualized with diaminobenzidine. The sections were counterstained with Mayer's hematoxylin.

### Statistic analysis

We compared the incidence of cancer with the Fisher's exact test.

## Results

**Figure 2:**
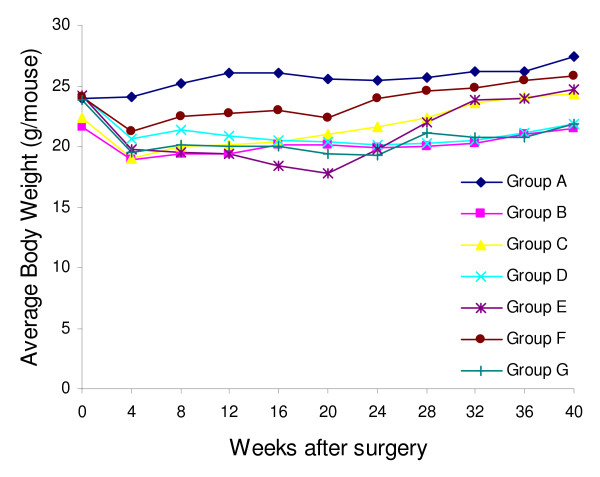
**Average body weight of A/J mice after surgery**. Non-operated control mice (Group A, -◆-) had the highest body weight.

Most mice (85%, 255/300) survived the surgery. The rest died of anesthesia, bleeding or unknown reasons during the surgery. After surgery, mice lost about 3 to 5 g of body weight, and then started to gain weight to a less extent compared to the non-operated control mice (Figure 2). At the end of the experiment, mice with reflux had slightly lower body weight than the non-operated control. Fifty three mice were excluded from this study. Among them, 16 were sacrificed due to sickness and 37 died before the end of experiment (11 due to blockage of the gastrointestinal tract, 4 due to infection subsequent to iron injection, 22 due to unknown reasons). Autopsy of these 22 mice failed to find any noticeable abnormalities probably due to decay of the bodies.

**Figure 3:**
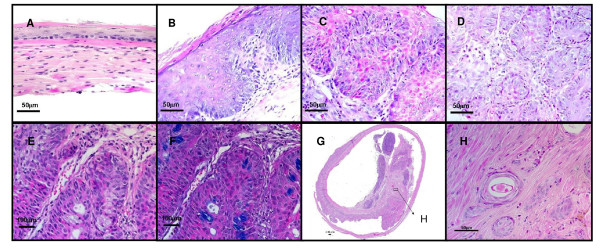
**Histopathology of mouse esophagus after reflux surgery**. (A) In the non-operated control group, the basal layer of the epithelium was smooth and the nuclei were in a single line. (B) The epithelium responded to surgery-induced reflux with hyperplasia. Layers of the squamous epithelium increased and papillae were enlarged. (C) After long-term reflux, the epithelial cells started to lose their polarity with condensed nuclei and increased mitosis. (D) Later on, the squamous epithelium lost its normal architecture. Dysplastic cells penetrated the basal membrane and invade into the stroma. (E) At 20 weeks after the surgery, mucin-producing cells were observed in the parabasal layer of the squamous epithelium. (F) Alcian blue staining confirmed mucin secretion in these scattered mucinous cells. (G, H) At 80 weeks after surgery, squamous cell carcinoma was observed in the Swiss-rolled esophagus of a mouse in Group B. Panel H is magnification of part of Panel G.

Normal mouse esophagus is covered by stratified keratinized squamous epithelium consisting of several layers of squamous epithelial cells (Figure 3A). Reflux surgery induced hyperplasia of the squamous epithelium with infiltration of inflammatory cells in the epithelium and submucosa of all the mice (Figure 3B). At week 40 and 80, some mice developed squamous dysplasia and squamous cell carcinoma (Figure 3C, D, G and 3H).

At 20 weeks after surgery, one esophageal sample with metaplasia was found each in wild-type mice (Group B) and *p53*^*A*135*V *^transgenic mice (Group C). Metaplasia was confirmed by Alcian blue staining as scattered mucinous cells in the middle of hyperplastic squamous cells (Figure 3E and 3F), as previously described in our rat model [31]. Mature goblet cells were not observed. The body weights of omeprazole treatment group (Group E) were significantly lower than the control group (data not shown).

At 40 weeks after surgery, we found 6, 2 and 1 esophageal samples with metaplasia in wild-type mice (Group B), *p53*^*A*135*V *^transgenic mice (Group C), and reflux plus gastrectomy with iron supplementation (Group G). Squamous cell carcinoma was found in one *INK4a/Arf*^+/- ^mouse (Group C) and 3 wild-type mice treated with reflux plus gastrectomy with iron supplementation (Group G). The incidence of squamous cell carcinoma in mice treated with reflux plus gastrectomy with iron supplementation (Group G) was significantly higher than that of mice treated with reflux alone (Group B) (p < 0.05).

Because we did not find any esophageal adenocarcinoma at week 40, we decided to give iron supplementation to the mice left in surgical control group (Group B). At 80 weeks after surgery, we found 12 mice with esophageal squamous cell carcinoma in 13 wild-type mice. All squamous cell carcinoma were located at the distal part of the esophagus and invaded into the muscle layer (Figure 3G). Under higher magnification, neoplastic cells appeared to be originated from squamous epithelial cell, and were surrounded by muscle fibers (Figure 3H). The only one mouse without squamous cell carcinoma had mild squamous dysplasia. Metaplasia was not observed in these mice (Table 1).

Unexpectedly, none of the mice developed either esophageal adenocarcinoma or typical intestinal metaplasia as rats in our previous studies [31].

### p53 expression in mouse esophagus

**Figure 4:**
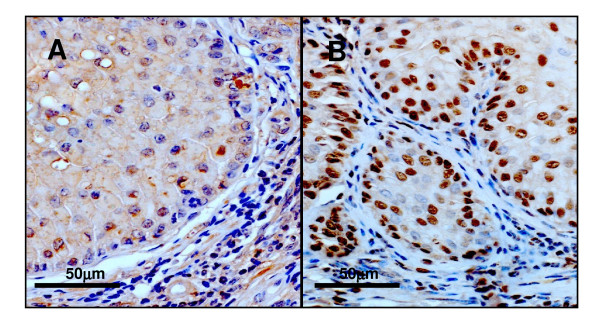
**p53 expression in the esophagi of wild-type and *p53*^*A*135*V *^mice**. Strong nuclear accumulation appeared in the esophageal epithelial cells of *p53*^*A*135*V *^mice, suggesting the mutant form of p53 protein.

In order to examine the expression pattern of mutant p53 protein in the esophageal epithelium, we immunostained p53 using a polyclonal p53 antibody. In the esophageal epithelium of wild-type mice slight background staining was observed. In contrast, strong nuclear staining was observed in the basal cells of esophageal epithelium of the *p53*^*A*135*V *^transgenic mice, suggesting accumulation of mutant p53 protein (Figure 4).

## Discussion

This study was primarily aimed to develop a surgical model of esophageal adenocarcinoma in mice. Reflux surgery was performed on wild-type, *p53*^*A*135*V *^transgenic and *INK4a/Arf*^+/- ^mice of A/J background. In addition, omeprazole (1,400 ppm in diet), iron (50 mg/kg/m, *i.p*.), or gastrectomy plus iron, were given to some of these mice in order to promote disease progression. Unexpectedly, we only observed metaplasia as scattered mucinous cells, but not typical intestinal metaplasia, in a small percentage of mice. Moreover, squamous cell carcinoma, but not esophageal adenocarcinoma, was induced.

Long-term gastroesophageal reflux in combination with iron (Group B at week 80) produced a high incidence of squamous cell carcinoma (92.3%, 12/13) in this study. Although gastroesophageal reflux has been often associated with Barrett's esophagus and esophageal adenocarcinoma, its association with squamous cell carcinoma has also been well documented in the literature. It is known that patients after total or partial gastrectomy had increased risk of developing esophageal and laryngeal squamous cell carcinoma [32-35]. Reflux either directly induced or promoted esophageal squamous cell carcinoma following carcinogen treatment in rat models [36,37]. In a previous study using the esophagoduodenal anastomosis procedure in rats, reflux increased the incidence of esophageal squamous cell carcinoma induced by 2,6-dimethylnitrosomorpholine or methyl-n-amylnitrosamine by 40% [14]. Nevertheless, it was interesting why A/J mice were not susceptible to esophageal adenocarcinoma after reflux surgery. It is possible that mouse esophageal epithelium might respond to gastroesophageal reflux differently from rat esophageal epithelium, even though they are very similar in histology. Although the exact underlying mechanism remains puzzling, genetic factors may play a critical role.

Scattered mucinous cells were observed in mouse esophagi after surgery, suggesting that induction of Barrett's esophagus and esophageal adenocarcinoma in mouse esophagus is still possible [31]. Our recent study on the rat model and human Barrett's esophagus have suggested squamous de-differentiation (i.e., loss of squamous transcription factors, p63, sox2) and columnar differentiation (i.e., gain of intestinal transcription factors, Cdx1, Cdx2, GATA4, HNF1α) were two essential aspects of intestinal metaplasia [38]. Since embryonic esophageal epithelium of *p63 *knockout mice and hypomorphic *sox2 *mice showed metaplastic changes of morphology and gene expression [39,40], we speculate that *p63 *or *sox2 *knockout mice may be more susceptible to Barrett's esophagus and esophageal adenocarcinoma after surgery. It is likely that proper combinations of genetic modifications and reflux surgery may be needed to induced Barrett's esophagus and esophageal adenocarcinoma in mouse esophagus.

Rodent models of esophageal adenocarcinoma have its inherent limitations. Rodents have keratinized squamous epithelium without submucosal glands in the esophagus, whereas humans have non-keratinized squamous epithelium with submucosal glands. When histological and physiological resemblance to humans is considered, a model with pigs may offer many advantages over rodent models. As humans, pigs have non-keratinized stratified squamous epithelium and submucosal glands in their esophagi [41]. Pigs may develop GERD and stress ulceration of the esophagus [42]. Pigs are also well suited for genetic modifications and surgery [43]. With endoscopy, pig esophagus may provide plenty of tissue samples for analysis. A pig model of Barrett's esophagus and esophageal adenocarcinoma is currently under development in our laboratory.

## Conclusion

In conclusion, reflux surgery induced esophageal squamous cell carcinoma, but not esophageal adenocarcinoma, in wild-type, *p53*^*A*135*V*^transgenic and *INK4a/Arf*^+/- ^A/J mice. Further studies are needed in order to develop a mouse model of esophageal adenocarcinoma.

## Abbreviations

GERD: gastroesophageal reflux disease.

## Competing interests

The authors declare that they have no competing interests.

## Authors' contributions

JH performed surgery on mice, contributed to study design, histopathology and data interpretation, and drafted the manuscript with XC. BL assisted surgery and animal care, and histopathology. CSY contributed to study design and data interpretation. XC performed surgery on mice, contributed to study design, histopathology and data interpretation, and drafted the manuscript with JH. All authors read and approved the final manuscript.

## Pre-publication history

The pre-publication history for this paper can be accessed here:

http://www.biomedcentral.com/1471-230X/9/59/prepub
